# P-1516. Addition of Single-Dose Aminoglycosides to Empiric Beta-Lactam Therapy in Septic Shock in a Setting with High Antimicrobial Resistance Rates

**DOI:** 10.1093/ofid/ofae631.1685

**Published:** 2025-01-29

**Authors:** Allison M Johnson, Marco R Scipione, Jing Zhao, Shannon Olson, Alex Huang

**Affiliations:** Detroit Medical Center, Detroit, Michigan; Detroit Receiving Hospital, Detroit, Michigan; Detroit Medical Center-Harper Hospital, Detroit, Michigan; Sinai-Grace Hospital Detroit Medical Center, Detroit, Michigan; Detroit Medical Center, Detroit, Michigan

## Abstract

**Background:**

At the Detroit Medical Center (DMC), the addition of an aminoglycoside (AMG) to β-lactam therapy is recommended in patients with septic shock due to high resistance rates; however, studies assessing the benefit of such combinations have yielded conflicting results. The objective of this study is to compare the outcomes of patients with septic shock who receive empiric β-lactam therapy with and without an AMG.

**Figure 1.**
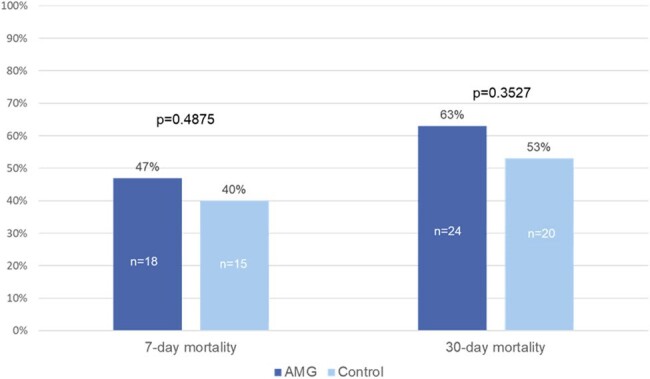
Mortality Outcomes

**Methods:**

This is a retrospective cohort study from 5/2021 to 7/2023. Patients were included if they met the following: 2 of 4 SIRS criteria, received vasopressors, serum lactate > 2 mmol/L, and appropriate empiric antimicrobial therapy within 24 hrs of admission . Patients who received an AMG within 24 hrs of admission were included in the AMG cohort, and those who did not were included in the control group. Key exclusion criteria were COVID-19, burn injury, and transfer to hospice care within 4 days of admission. The primary outcome was all-cause mortality at 7 days ; secondary outcomes included 30-day mortality, vasopressor-free days by day 7, ventilator-free days by day 28, and nephrotoxicity by day 7.

**Table 1.**
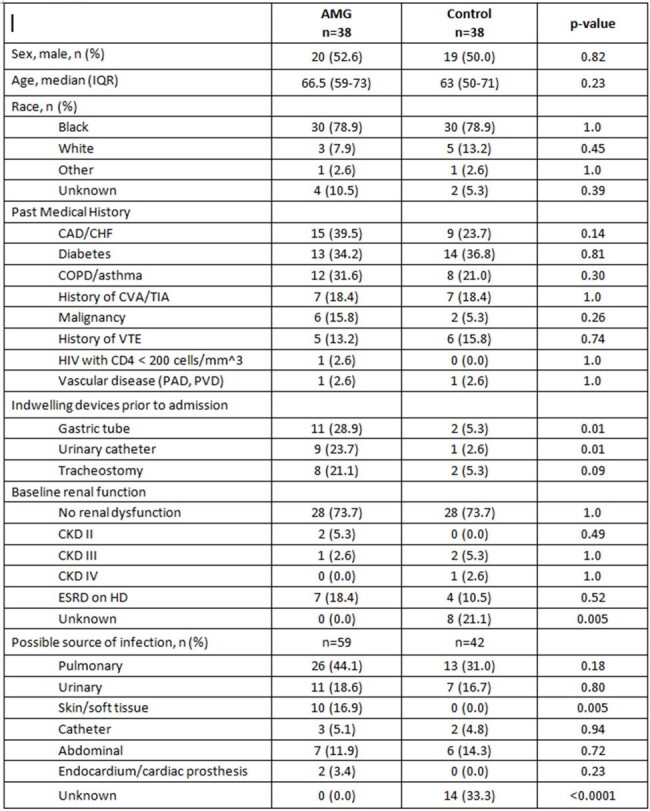
Patient and treatment characteristics

**Results:**

Seventy-six patients were included (AMG=38; Control=38). The most common source of infection in AMG and control groups was pulmonary (44% vs. 31%, p=0.18). More AMG patients had a presumed SSTI source (17% vs. 0.0%, p=0.0049) patients, positive blood culture (79% vs. 26%, p< 0.0001), prior history of MDRO pathogen (22% vs. 0.0%, p=0.003), and received meropenem as initial empiric therapy (40% vs. 3%, p< 0.0001). APACHE II scores were similar (30 vs. 28, p=0.09). Time to appropriate antibiotic therapy was 4.8 hrs in the AMG group vs. 6.4 hrs in the control group, p=0.81. There were no differences in 7-day mortality (47% vs. 40%, p=0.49), 30-day mortality (63% vs. 53%, p=0.3527), vasopressor-free days by day 7 (2 vs. 2, p=0.08), or ventilator-free days by day 28 (2.5 vs. 8.5, p=0.31). There were also no differences in nephrotoxicity by day 7 (50% vs. 67%, p=0.23).

**Table 1.**
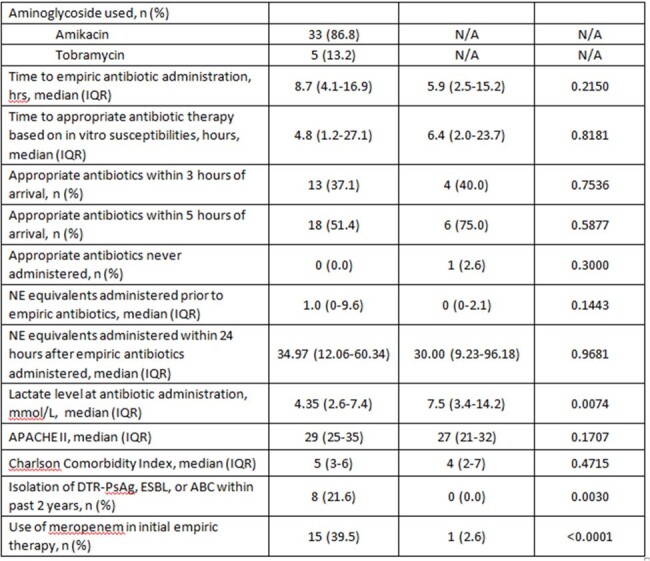
Patient and treatment characteristics, continued

**Conclusion:**

Further evaluations should be conducted to determine whether an AMG in combination with β-lactams should be used in patients with septic shock.

**Table 2.**
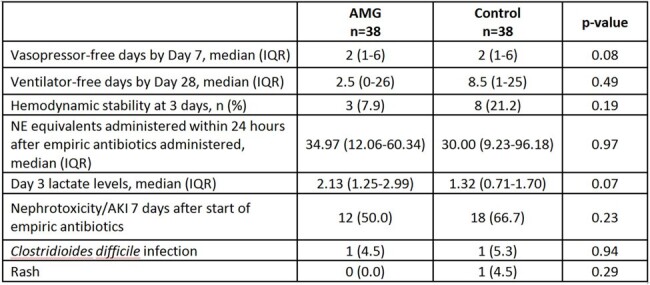
Other clinical outcomes

**Disclosures:**

**All Authors**: No reported disclosures

